# The characteristic findings of an inverted-type discoid lateral meniscus tear: a hidden tear pattern

**DOI:** 10.1186/s12891-019-2618-9

**Published:** 2019-05-17

**Authors:** Kengo Shimozaki, Junsuke Nakase, Yasushi Takata, Kazuki Asai, Kazu Toyooka, Katsuhiko Kitaoka, Hiroyuki Tsuchiya

**Affiliations:** 1 Department of Orthopaedic Surgery, Graduate School of Medical Sciences, Kanazawa University, 13-1 Takara-machi, Kanazawa-shi, Ishikawa-ken 920-8641 Japan; 2 Department of Orthopedic Surgery, Kijima Hospital, Kanazawa, Japan

**Keywords:** Inverted-type tear, Discoid lateral meniscus, Magnetic resonance imaging, Arthroscopic partial meniscectomy, Trauma

## Abstract

**Background:**

The purpose of this study was to reveal the clinical history and physical and magnetic resonance imaging (MRI) findings of patients with an inverted-type discoid lateral meniscus (DLM) tear compared with those with a symptomatic and torn discoid meniscus without inverted tear patterns.

**Materials and methods:**

Between 2014 and 2016, 12 patients underwent arthroscopic partial meniscectomy for an inverted-type DLM tear (inverted group). We age-matched these patients with 12 controls who were extracted from many normal DLM tear cases in the same period (non-inverted group). The assessment items were traumatic history with the onset of pain, the mean duration between the appearance of symptoms and surgery, preoperative knee range of motion (ROM), positive findings on the McMurray test, knee locking or catching, and characteristic MRI findings. These items were compared between the two groups using χ^2^ and Student’s t-tests.

**Result:**

All patients in the inverted group had clear trauma with the onset of pain during sports or daily life activities, and 7 of the 12 patients with a non-inverted type of DLM tear had clear trauma. There was a significantly higher rate of traumatic history in the inverted group than in the non-inverted group (*P* = 0.03). The mean duration between the appearance of symptoms and surgery, preoperative knee ROM, positive findings on the McMurray test, and knee locking or catching were not significantly different between the inverted and non-inverted groups. On MRI, the diagnosis ratio of DLM was significantly higher in the non-inverted group (9/12 cases) than in the inverted group (3/12 cases, *P* = 0.04). Nine of the 12 inverted-type patients had the characteristic findings of an inverted-type DLM tear, including a duplicated or enlarged posterior horn and blunting of the inner rim, on the sagittal plane of an MRI.

**Conclusion:**

Patients with inverted-type DLM tears had clear trauma and infrequently had the characteristic MRI findings that are observed in patients with normal DLM tears. Physicians should suspect that an inverted-type DLM tear is present during diagnosis and focus on the posterior horn to find the inverted sign on the MRI sagittal plane.

**Level of evidence:**

Level III.

## Background

The discoid lateral meniscus (DLM) of the knee is an abnormally wide and thick meniscus that completely or incompletely covers the articular surface of the lateral tibial plateau. It was first described by Young in 1889 [1]. The incidence of DLM tear is reported to be between 0.4 and 16% [2], with more than 10% in Asia [3, 4], but the origin of this condition is uncertain. Previous reports indicated that the central portion of the DLM is subjected to shear stress and is easily damaged by repeated minor trauma because of abnormal collagen fibre patterns [5–7]. The tear patterns in the lateral meniscus have been categorised by Dandy [8]. The characteristic physical findings, imaging findings, and treatment methods of each type of tear are also widely known. However, the inverted-type DLM tear has not been described in detail. LaMont et al. [9] only reported a case control series about the characteristics of inverted-type DLM tears. They reported that a discoid meniscus with an inverted segment does not have the standard radiographic and arthroscopic features that are normally associated with a discoid meniscus. Therefore, it is important to know its features in order to prevent misdiagnosis.

The present study aimed to reveal the clinical history and physical and magnetic resonance imaging (MRI) findings of patients with an inverted-type DLM tear compared with those with a symptomatic and torn discoid meniscus without an inverted tear pattern.

## Materials & methods

This study was approved by the Kanazawa University Medical Ethics Review Committee (approval No. 1842–1). Written informed consent was obtained from all patients included in this study.

**Fig. 1:**
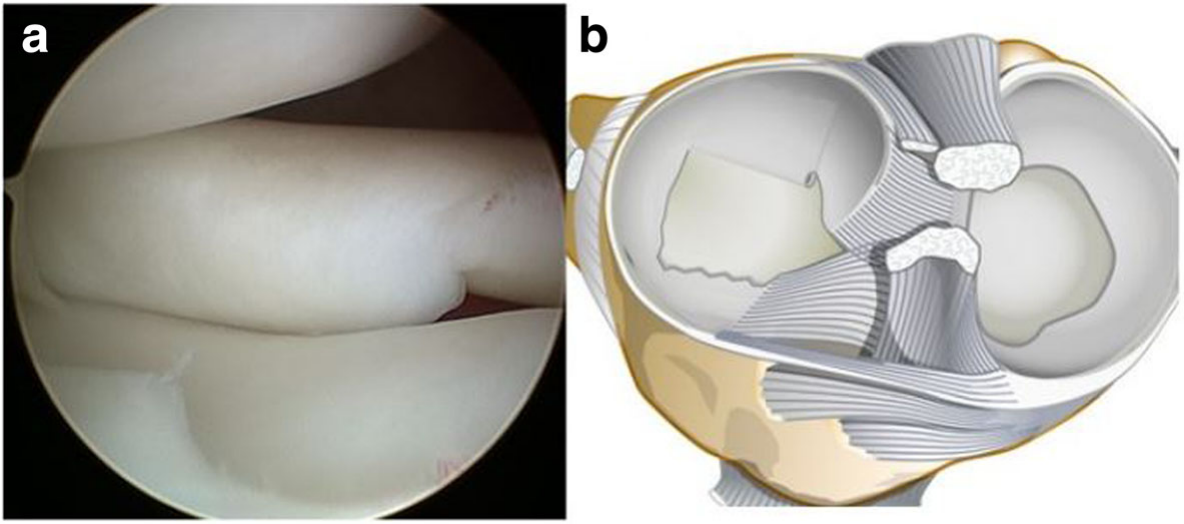
Inverted-type discoid lateral meniscus tear. Arthroscopic findings (**a**). The torn portion was inverted below the posterior horn. Schema of the tear (**b**) only in the anterior part; there were no posterior peripheral rim tears and instability of the posterior horn

Between January 2014 and September 2016, 12 patients underwent arthroscopic partial meniscectomy for an inverted-type DLM tear (inverted group), which included both complete and incomplete types of tears. To investigate the characteristic findings of patients with inverted-type DLM tears, 12 controls who were age-matched with patients in the inverted group were extracted from normal DLM tear cases (non-inverted group). All of the patients in both groups had knee swelling and lateral knee joint pain during exercise; therefore, they underwent MRI. We diagnosed both the inverted and non-inverted types of tears using arthroscopic findings. The average ages of the patients at the time of surgery were 19.6 ± 5.7 years (range: 15 to 33 years) and 17.3 ± 8.1 years (range: 10 to 43 years) in the inverted and non-inverted groups, respectively. An inverted-type DLM tear was defined arthroscopically as a torn portion of the central discoid body that was inverted beneath the intact posterior peripheral horn of the lateral meniscus (Fig. 1a-b). An experienced orthopaedic surgeon (K.K.) performed all operations at our institution. Patients’ traumatic history with the onset of pain, the mean duration between the appearance of symptoms and surgery, preoperative knee range of motion (ROM), positive findings on the McMurray test, knee locking or catching, and MRI findings to determine if the tear fulfilled the diagnostic criteria for a DLM tear were retrospectively assessed. The characteristic findings of inverted-type tears were assessed to determine which of these findings supported the diagnosis of an inverted-type DLM tear.

### MRI procedures

MRI was performed with a dedicated knee coil on a 0.4 T unit (APERTO, Hitachi Medical Corporation, Tokyo, Japan). Each patient was fixed in the supine position with the knee joint in mild flexion. A T2-weighted image in the coronal and sagittal planes was used to evaluate the characteristic MRI findings. The section thicknesses of the coronal and sagittal views were 3.0 mm. The interval gaps for both views were 0.5 mm.

**Fig. 2:**
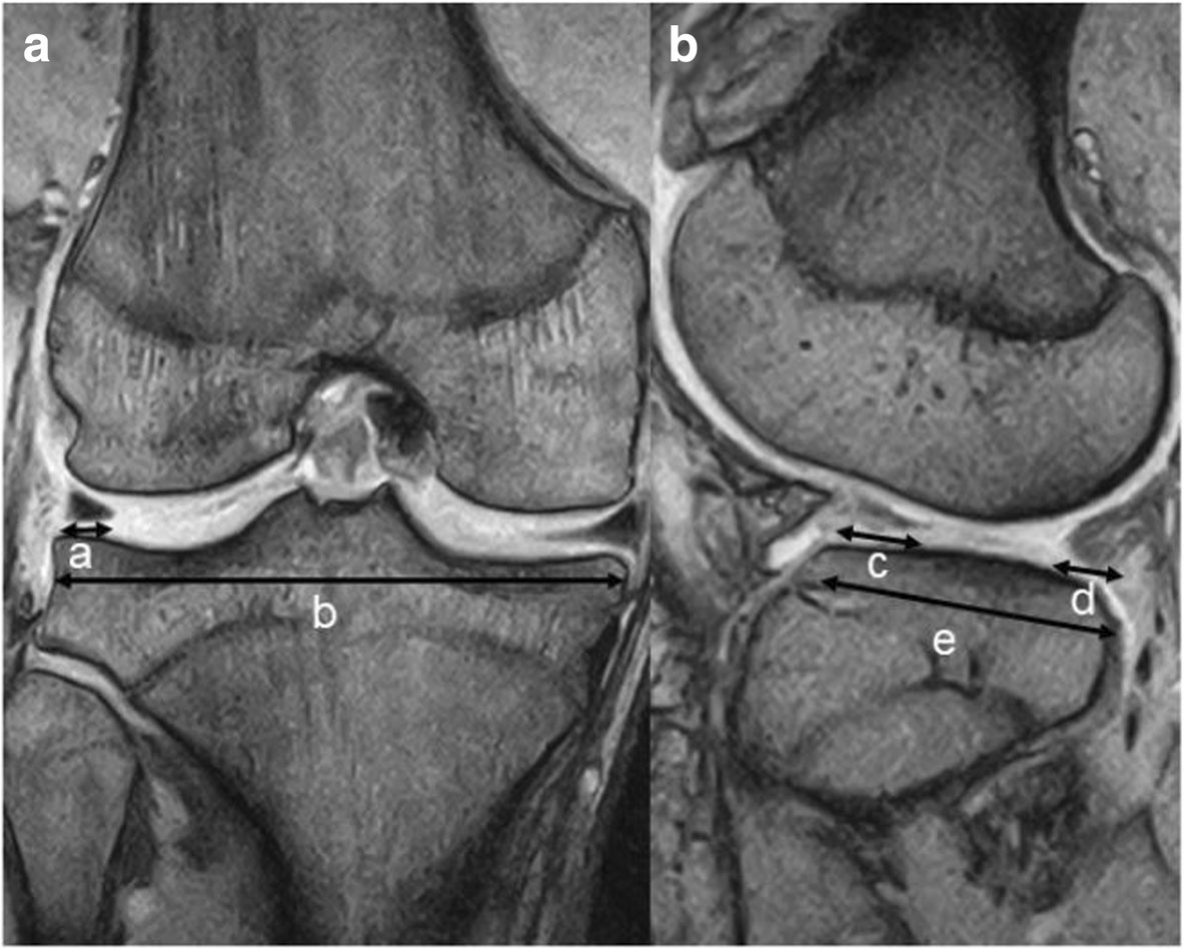
Diagnostic parameters of the DLM on an magnetic resonance imaging scan. Coronal plane (**a**). Lateral meniscal width; a mm. Ratio of the meniscus to the tibia; a/b × 100%. Sagittal plane (**b**). Percent coverage of the meniscus; c + d/e × 100%. DLM: discoid lateral meniscus

On an MRI scan, the diagnostic criteria for a DLM tear were based on those from a study by Samoto et al. [10] that showed very high sensitivity and specificity for both torn and non-torn DLMs. Therefore, we measured four parameters. The first parameter was the lateral meniscal width (LMW) [11]. In the coronal plane of an MRI scan at the central part of the anterior-posterior diameter of the tibia, an LMW (a) larger than 15 mm indicates a DLM tear (Fig. 2a). The second parameter was the ratio of the meniscus to the tibia in the coronal plane. Using the same plane that was used to measure the LMW, we calculated the ratio of the LMW to the maximum tibial width according to the formula a/b × 100% (Fig. 2a), and we determined that a ratio greater than 20% was regarded as a positive finding. The third parameter was the percent coverage of the meniscus in the sagittal plane. On the MRI scan in the sagittal plane at the middle of the lateral femoral condyle, we determined the ratio of the sum of the anterior and posterior segments of the lateral meniscus to the width from the anterior horn to the posterior horn according to the following formula: c + d/e × 100% (Fig. 2b), and we considered that a ratio greater than 75% was regarded as a positive finding. The fourth parameter was continuity of the anterior and posterior horns in the coronal plane. The number of consecutive sagittal slices showing continuity between the anterior and posterior horns of the meniscus was counted [12]. We considered that more than five slices indicate a DLM tear.

**Fig. 3:**
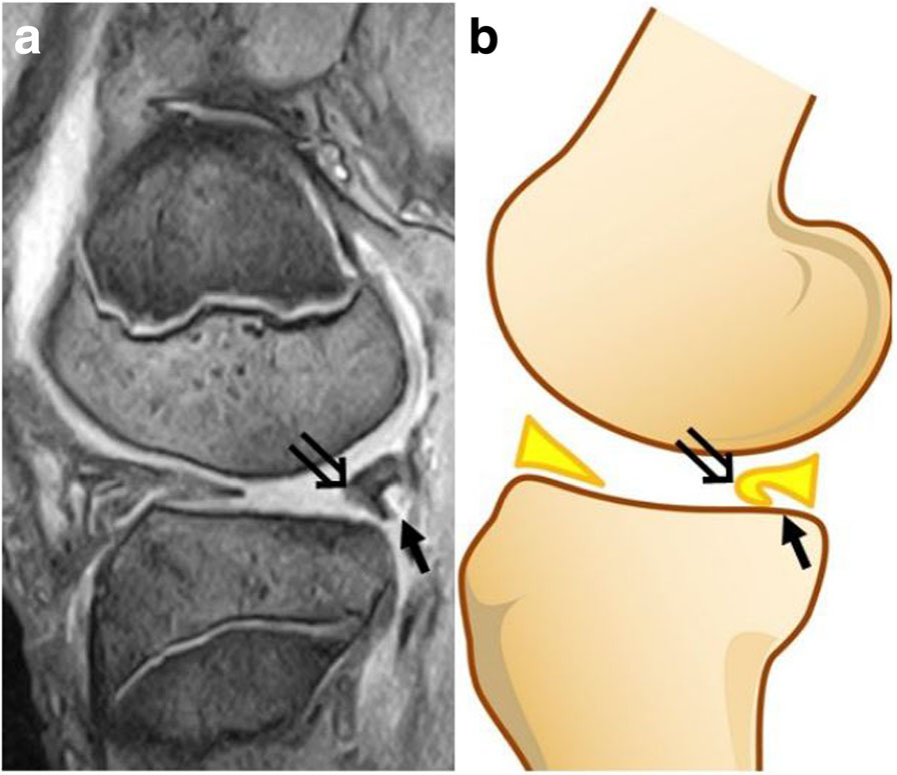
Inverted sign seen on the magnetic resonance imaging scan in the sagittal plane. The posterior horn was duplicated (→), and the inner rim of the posterior lateral meniscus was blunted (⇒) (**a**). Schema of the inverted sign (**b**)

Although each of the four diagnostic criteria alone might be reliable for diagnosis, we diagnosed a DLM tear when two or more of the criteria were satisfied to reduce error due to the measurement conditions. We considered that the characteristic findings to support the diagnosis of an inverted-type tear were a duplicated or enlarged posterior horn and blunting of the inner rim in the sagittal plane, and we named these findings the inverted sign (Fig. 3a-b).

The MRI findings were retrospectively interpreted by two experienced orthopaedic surgeons in a blinded fashion regarding the patients’ names and arthroscopic findings. Then, the MRI findings were evaluated twice at intervals of about 2 weeks. When the judgments of the two surgeons differed, a third experienced orthopaedic surgeon determined the result.

### Statistical analyses

The data were analysed using the Statistical Package for the Social Sciences for Windows (version 23.0; SPSS Inc., Chicago, IL, USA). The assessment items of the inverted and non-inverted groups were compared using a χ^2^ test for a history of trauma, the McMurray test, knee locking or catching, and MRI findings; and Student’s t-test was used to determine the mean duration between the onset of trauma and surgery, as well as knee ROM. The inter-class and intra-class reliabilities were assessed using the intra-class correlation coefficient (ICC). The level of significance for all statistical analyses was set at α = 0.05.

## Results

The patients’ demographic characteristics and clinical data are presented in Table 1. All patients in the inverted group had clear trauma with the onset of pain during a sports activity or activities of daily life, and 7 of the 12 patients in the non-inverted group had clear trauma. There was a significantly higher rate of traumatic history in the inverted group than in the non-inverted group (*P* = 0.03). The mean duration between the appearance of symptoms and surgery, preoperative knee ROM, positive findings on the McMurray test, and knee locking or catching were not significantly different between the inverted and non-inverted-type groups. The diagnostic rate of DLM tear using MRI was significantly higher in the non-inverted group (9 of 12 patients) than in the inverted group (3 of 12 patients, *P* = 0.04). Nine of the 12 inverted-type patients had an inverted sign on the MRI scan in the sagittal plane. The characteristic findings were a duplicated or enlarged posterior horn and a blunted inner rim in the sagittal plane. In contrast, none of the participants in the non-inverted group had an inverted sign. For all MRI findings, the ICC for the intra-class reliability was 0.925, and the ICC for the inter-class reliability was 0.901.

**Table 1:** Patients’ demographic characteristics and clinical data

	Inverted group (*n* = 12)	Non-inverted group (*n* = 12)	*P*-value
History of trauma (cases)*	12	7	0.03***
Positive findings of the McMurray test (cases)*	4	5	0.68
Positive findings of locking or catching (cases)*	1	3	0.59
Diagnosis of DLM tear by MRI (cases)*	3	9	0.04***
“Inverted sign” (cases)*	9	0	< 0.05***
Knee extension angle (°)**	−2.7 ± 5.1	−1.6 ± 3.2	0.56
Knee flexion angle (°)**	128.7 ± 23.3	132.9 ± 25.4	0.68
Mean duration between the appearance of symptoms and surgery (days)**	64.1 ± 72.4	50.9 ± 48.7	0.60

*DLM* discoid lateral meniscus, *MRI* magnetic resonance imaging; inverted sign: a duplicated or enlarged posterior horn and blunting of the inner rim in the sagittal plane detected by MRI

*The data and *P*-values were determined using the χ^2^ test

**The results are presented as the mean ± standard deviation, and *P*-values were determined with Student’s t-test

***Significant difference

## Discussion

In our study, the participants in the non-inverted group had a significantly lower rate of traumatic history than those in the inverted group. Although there was no significant difference between the groups, the mechanical symptoms and McMurray test tended to be positive in participants in the non-inverted group compared with those in the inverted group. In contrast, the mean duration between the appearance of symptoms and surgery tended to be longer in the inverted group than in the non-inverted group, despite the occurrence of clear trauma and swelling in all inverted-type patients. Patients with a symptomatic DLM with or without tears, especially when young, generally do not have a history of trauma with the onset of pain, and often have mechanical symptoms such as knee locking or catching, as seen on the McMurray test [13, 14].

These results were similar to those of LaMont et al. [9]; they reported that in 19 patients who had an inverted segment, 18 had a clear history of trauma, 11 experienced knee swelling, and all patients had lateral knee pain during exercise. However, they reported that mechanical symptoms and a positive McMurray test were present in only four of the 19 patients. This may be due to the characteristics of inverted-type DLM tears. In the inverted type of DLM tear, the tear occurs only in the anterocentral part of the meniscus, and the torn portion forms a large flap. There were no peripheral tears or instability of the posterior horn. Therefore, once the torn flap of the central discoid body is inverted and moved into the intact posterior horn, its position will be stabilised and it will not move, even during exercises such as extension and flexion of the knee. In fact, in the arthroscopic findings of this study, the torn inverted portion did not move at all during extension and flexion of the knee. Therefore, probing was necessary to expose the torn inverted portion. Because the position of the torn portion was stable, the symptoms were not severe and it was possible for the patient to continue exercising. In addition, mechanical symptoms and the McMurray test, which focus on dynamic motion of the meniscus, were not clear, and the duration of time between the appearance of symptoms and surgery tended to be longer for patients with inverted-type DLM tears than for patients with non-inverted types of DLM tears.

Because the torn flap of the central discoid body is inverted beneath the intact posterior horn, an ‘O’ shape resembling a normal lateral meniscus is observed. Therefore, few features indicate a DLM tear, and it is difficult to diagnose it using MRI. Nine patients had a duplicated or enlarged posterior horn and a blunted inner rim in the sagittal plane, as seen on MRI scans, which we named the inverted sign. This characteristic finding is useful for diagnosing an inverted-type DLM tear. Two of three patients who did not have the characteristic MRI findings of an inverted-type tear but showed mechanical symptoms and had a positive McMurray test were diagnosed as having a DLM tear by MRI. In addition, they had a smaller amount of meniscal inversion than other patients did, as seen on arthroscopy. This partly explains why we could not diagnose these patients with an inverted-type DLM tear. The characteristic physical and MRI findings may be related to the size of the torn inverted portion.

**Fig. 4:**
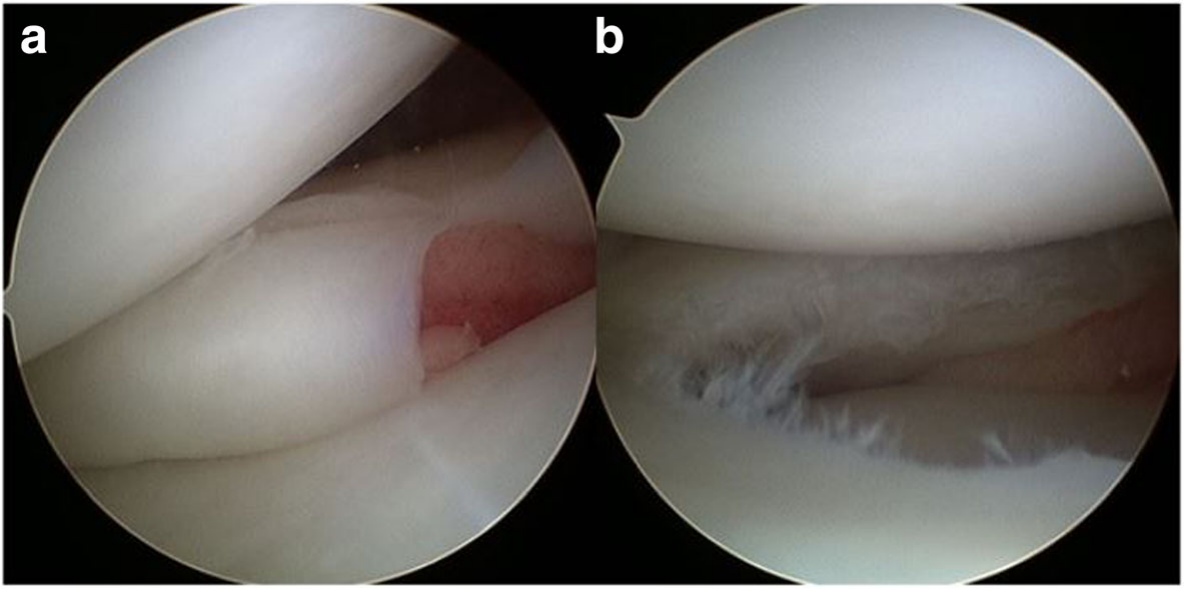
Arthroscopic findings of a representative patient. The inverted-type discoid lateral meniscus tear before (**a**) and after (**b**) treatment. Because there were no posterior peripheral rim tear and instability of the posterior horn, only the flap portion was resected

The traditional treatment for a symptomatic DLM tear is total or subtotal meniscectomy [3, 15]. Although some studies have reported good long-term clinical results, radiographically, many studies showed a high rate of early degenerative changes of the lateral compartment of the knee [16, 17]. Therefore, a recently recommended treatment plan for a symptomatic DLM tear is arthroscopic meniscal reshaping using partial meniscectomy, with or without meniscal repair [18, 19]. None of the patients in our study with inverted-type DLM tears had a posterior peripheral rim tear or instability of the posterior horn, but the inverted portion of the central discoid body was torn. Therefore, we performed only resection of the flap portion, which was reduced using a probe, without meniscal repair (Fig. 4a-b). This procedure is called meniscal reshaping or plastic meniscectomy.

The inverted-type DLM tear has two diagnostic points. The first is the patient’s medical history, especially traumatic history. Even if physical findings do not suggest the presence of a meniscal lesion, physicians should suspect a lateral meniscus injury in patients with a clear history of trauma or knee swelling and lateral knee pain during exercise. The second point is to find the inverted sign on the sagittal plane of an MRI scan. It is important to carefully interpret MRI findings while suspecting the presence of an inverted-type tear when the physical examination is poor. There are some benefits to surgeons to knowing these diagnostic points. These diagnostic points are useful in determining the treatment strategy and timing of operation. Because there are few physical symptoms and only partial meniscectomy is often performed during surgery, it is possible to select the surgical timing and infer the timing of return to sports among athletes. These are also useful in preventing missing of meniscus tears during arthroscopy. It is reported that the inverted-type DLM tear looks like a normal lateral meniscus without a meniscus tear at first glance and that probing is necessary to expose the inverted portion [9]. Thus, missing a meniscus tear can be prevented through prior knowledge of an inverted-type DLM tear and its diagnostic points.

One of the strong points of our study is that the inverted-type tear is a rare and hidden tear pattern of the DLM. It is possible to predict the treatment strategy before surgery by knowing the type of DLM tear. In addition, in all patients, we confirmed the tear pattern using arthroscopy. However, this study has some limitations. First, we used a 0.4 T MRI apparatus in this study, which does not have good quality compared with other recent types of MRI apparatuses. It is thus necessary to improve the quality in future studies. Second, the study was retrospective, and bias was generated when we interpreted the MRI scans. Therefore, we measured the data twice in a blinded method regarding the patients’ names and arthroscopic findings. Last, the sample size of this study was small. Inverted-type discoid lateral meniscus tears are not common or well known. Although the characteristics of inverted-type DLM tears could be shown by including a control group in this study, further research on this topic is required.

## Conclusions

Patients with inverted-type DLM tears had clear trauma and infrequently had the characteristic MRI findings of normal DLM tears. The inverted type of DLM tear should be suspected during diagnosis of the condition, and physicians should focus on the posterior horn to find the inverted sign on the MRI sagittal plane.

## Abbreviations

DLM: Discoid lateral meniscus
ICC: Intra-class correlation coefficient
LMW: Lateral meniscal width
MRI: Magnetic resonance imaging
ROM: Range of motion
